# P-1301. Pilot Study Utilizing Wastewater-Based Epidemiology to Determine Presence of Gram-Negative Enterobacterial Targets in Southwest Virginia Sewersheds

**DOI:** 10.1093/ofid/ofae631.1482

**Published:** 2025-01-29

**Authors:** Lia W Lehrer, Kathleen Linsenman, Kaylin Young, Lauren E Luther, Troy Stallard, Rachel L Rogers, Susan Tolliver, Sara Houser, Jayasimha Rao

**Affiliations:** Radford University Carilion, Roanoke, Virginia; Radford University Carilion, Roanoke, Virginia; Radford Univeristy ; Carilion Clinic, Salem, Virginia; Radford University Carilion, Roanoke, Virginia; Radford University, Roanoke, Virginia; Carilion Clinic; Radford University, Roanoke, Virginia; Carilion Clinic, Roanoke, Virginia; Radford University Carilion, Roanoke, Virginia; Carilion Clinic/Virginia Tech Carilion School of Medicine, Roanoke, Virginia

## Abstract

**Background:**

Wastewater-based epidemiology (WBE) detects and mitigates pathogen outbreaks by evaluating conditions in a specific community. Between January 2019 and September 2022, *Escherichia coli*, *Citrobacter freundii*, *Pseudomonas aeruginosa,* and *Salmonella enterica* comprised about one-third of the local health department’s reported healthcare-associated infections. This pilot study attempted to detect and quantitatively assess the prevalence of these bacterial pathogens in order to report public health data to the state health department, enabling effective outbreak preparedness and response.

Pathogen Standard Curves with Plotted Unknowns

**Figure:**
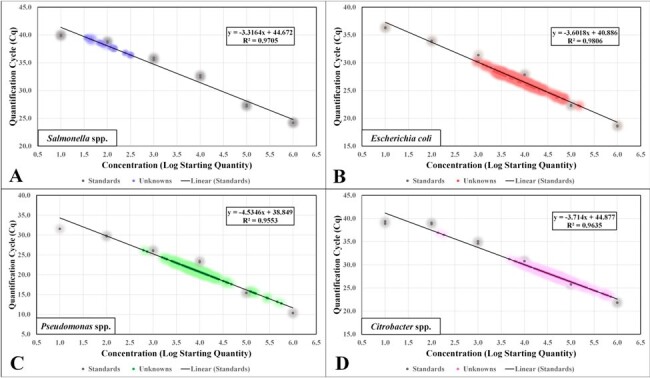

Depicted above are the standard curves with plotted unknowns from the 12 weeks of collection (n=83) for each organism. Each linear equation and R2 value is represented for the standards. A: Salmonella spp. standards and unknowns. B: E. coli standards and unknowns. C: Pseudomonas spp. standards and unknowns. D: Citrobacter spp. standards and unknowns.

**Methods:**

Wastewater samples (n=83, 100 mL) were collected from eight community watersheds in Roanoke and Salem, VA, including two healthcare facilities, over a 12-week period (February-May, 2023). Microbial total nucleic acids (TNAs) were extracted with the Promega Wizard enviro kit and quantified using a Nanodrop spectrophotometer. Bacterial DNA target detection was performed using gene-specific primers (*Salmonella* spp.: *invA, E. coli: gadA; Pseudomonas* spp.: 23S rRNA; *Citrobacter* spp.: *tpl*). TaqMan probe sets with unique fluorophores were selected with CFX Opus 96, and 16S rDNA was normalized with microbial population. Amplification efficiency was measured by using cycle quantification (Cq) value.

Pathogen Cq and Copies/L Averages

**Figure:**
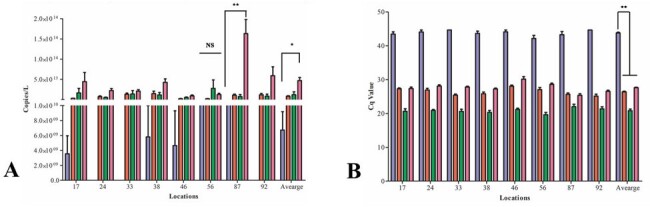

Each organism is depicted as follows according to color: purple, Salmonella spp.; red, E. coli; green, Pseudomonas spp.; and pink, Citrobacter spp.. A: Copies/L per organism for each location over 12 weeks. Average included for all locations. B: Cq per organism for each location over 12 weeks.

**Results:**

*E. coli* (average of Cq 26.6), *Citrobacter* spp. (Cq 28.3), and *Pseudomonas* spp. (Cq 20.8) were detected in all locations and not significantly different with copies/L. *Pseudomonas* spp. had the lowest Cq in most of the wastewater samples (Figure 1). *Salmonella* spp. (Cq 44.1) was identified sporadically in locations, such as hospital and influent collections (1.52 x 10^11^) copies/L, which is significantly different at (*P* < 0.001)(Figure 2). 16S rDNA was detected in most samples (Cq 16.3)(Figure 3).

Relative Abundance (%) of Microbial Population

**Figure:**
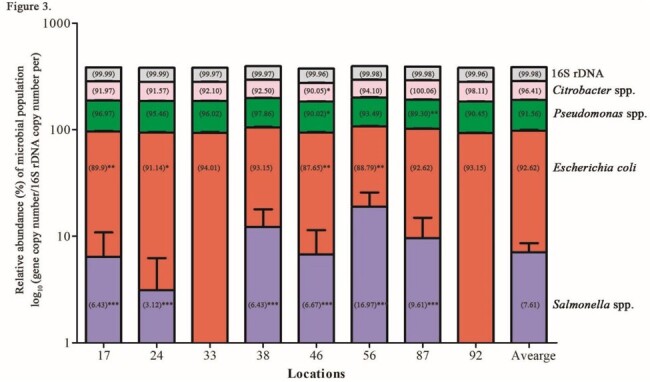

Relative abundance (%) of each organism is depicted by parenthesis. P-Values are represented as follows: *P < 0.05; **P < 0.1, ***P < 0.001.

**Conclusion:**

According to the CDC, WBE in conjunction with other data collection can provide real-time trends of a population to promote public health. WBE surveillance targeting the most prevalent pathogens in communities can be a vital tier to public health defense. Reported findings indicate the presence of prevalent bacterial pathogens in the local Roanoke and Salem, VA sewersheds.

**Disclosures:**

**All Authors**: No reported disclosures

